# Large‐scale validation of an improved and resource‐saving immunoprecipitation‐mass spectrometry assay for plasma amyloid‐β biomarkers in Alzheimer's disease

**DOI:** 10.1002/alz.092562

**Published:** 2025-01-09

**Authors:** Yijun Chen, Xuemei Zeng, Marcos Olvera‐Rojas, Anuradha Sehrawat, Tara K Lafferty, William E Klunk, Milos D Ikonomovic, Tharick Ali Pascoal, Kirk I. Erickson, Victor L Villemagne, Ann D Cohen, Nathan Yates, Irene Esteban‐Cornejo, Oscar L. Lopez, Beth E. Snitz, Sarah Berman, Robert Sweet, Thomas K Karikari

**Affiliations:** ^1^ University of Pittsburgh, Pittsburgh, PA USA; ^2^ Faculty of Sport Sciences, Sport and Health University Research Institute (iMUDS), University of Granada., Granada, Granada Spain; ^3^ University of Pittsburgh School of Medicine, Pittsburgh, PA USA; ^4^ AdventHealth, Orlando, FL USA; ^5^ University of Pittsburgh Alzheimer's Disease Research Center (ADRC), Pittsburgh, PA USA; ^6^ Instituto de Investigación Biosanitaria (ibs.GRANADA), Granada Spain; ^7^ University of Pittsburgh Alzheimer's Disease Research Center, Pittsburgh, PA USA; ^8^ Department of Psychiatry and Neurology, University of Pittsburgh, Pittsburgh, PA USA; ^9^ Department of Psychiatry, School of Medicine, University of Pittsburgh, Pittsburgh, PA USA

## Abstract

**Background:**

Amyloid β (Aβ) deposition in the brain is a pathological hallmark of Alzheimer's disease (AD). While immunoprecipitation‐mass spectrometry (IP‐MS) stands out as an accurate method for quantifying blood‐based Aβ peptides, its major limitations such as prolonged sample preparation, extensive analysis time, large specimen volume, and high costs, present opportunities for improvement. Consequently, we aimed to develop a novel plasma IP‐MS Aβ assay that employs simplified and significantly shorter analytical procedures, along with much‐reduced sample volumes.

**Method:**

We evaluated effects of reducing the sample volume, changing the buffers, implementing heavy labeled internal standards, and switching to a more cost‐effective MALDI‐Tof mass spectrometer versus the original Shimadzu IP‐MS Aβ assay (Nakamura et al., 2018 Nature). This led to an improved assay, referred to as the Pittsburgh IP‐MS Aβ assay. Analytical validation covered linearity, accuracy, precision, and recovery. In clinical validation, we compared the new and original Aβ assays for identifying Aβ‐PET positivity and cognitive impairment in two independent cohorts.

**Result:**

The Pittsburgh assay streamlined the immuno‐enrichment step with a novel binding buffer, cutting background noise and reducing processing time by 50%. Additionally, it allowed for potential sample volumes as low as 50 µl and decreased antibody and paramagnetic bead usage by 75% each. The assay utilized the cost‐friendly MALDI‐Tof instrument from Bruker, a departure from the AXIMA Performance platform used in the original assay.

The analytical validation showed consistent signal linearity, improved spike recovery with heavy labeled Aβ1‐40 or Aβ1‐42, but equivalent precision between the two IP‐MS methods. In the AGUEDA cohort (n=148), the Pittsburgh assay demonstrated better AUCs than the older method for identifying Aβ‐PET‐positive individuals. This trend persisted in the University of Pittsburgh ADRC cohort (n=30) when comparing accuracy to identify patients with clinical assessed AD positive.

**Conclusion:**

We have developed a more efficient and cost‐effective IP‐MS plasma Aβ assay, outperforming the original Shimadzu assay in clinical utility, while upholding consistently high analytical performance. The cost, time, and reagent savings along with the utilization of a more affordable and widely available instrument will empower more research laboratories to conduct IP‐MS analysis of Aβ in blood more effectively.

